# Synthesis and anthelmintic activity of aminochalcones against multiresistant *Haemonchus contortus*

**DOI:** 10.1590/S1984-29612025046

**Published:** 2025-10-17

**Authors:** Matheus Luiggi Freitas Barbosa, Andreza Pereira Braga, Karin Vitória Maria Mendonça Ferreira, Raphael Ferreira Oliveira, Rafaela da Silva Pereira, Letícia Oliveira da Rocha, Jaiza Maria Lima Dias, Jésyka Macedo Guedes, Hélcio Silva dos Santos, Joice Farias do Nascimento, Flavia Oliveira Monteiro da Silva Abreu, Wesley Lyeverton Correia Ribeiro, Lorena Mayana Beserra de Oliveira

**Affiliations:** 1 Universidade Estadual do Ceará – UECE, Faculdade de Veterinária, Laboratório de Doenças Parasitárias, Programa de Pós-graduação em Ciências Veterinárias – PPGCV, Fortaleza, CE, Brasil; 2 Universidade Estadual do Norte Fluminense Darcy Ribeiro – UENF, Centro de Biociências e Biotecnologia, Laboratório de Biologia Celular e Tecidual, Rio de Janeiro, RJ, Brasil; 3 Universidade Vale do Acaraú – UVA, Centro de Ciências Exatas e Tecnologia, Sobral, CE, Brasil; 4 Universidade Estadual do Ceará – UECE, Centro de Ciências e Tecnologia, Programa de Pós-graduação em Ciências Naturais, Fortaleza, CE, Brasil; 5 Universidade Federal do Ceará – UFC, Faculdade de Medicina, Departamento de Fisiologia e Farmacologia, Fortaleza, CE, Brasil

**Keywords:** Small ruminants, active compounds, structural modifications, antiparasitic effect, gastrointestinal nematodes, Pequenos ruminantes, compostos ativos, modificações estruturais, efeito antiparasitário, nematódeos gastrintestinais

## Abstract

The objective of this study was to synthesize and evaluate the *in vitro* activity of aminochalcones against *Haemonchus contortus* eggs and adults. Aminochalcones 1 and 2 were synthesized using Claisen–Schmidt condensation and characterized using Fourier-transform infrared spectroscopy, nuclear magnetic resonance, and mass spectrometry. The activity of both aminochalcones was assessed in the egg hatch test and that of aminochalcone 1 was further evaluated in the adult worm motility test using multiresistant *H. contortus*. The chemical structures of the synthesized compounds were confirmed, and changes induced in eggs and adults were assessed using scanning electron microscopy (SEM). Aminochalcones 1 and 2 inhibited larvae hatching by 98.70 and 99.89%, respectively, at concentrations of 0.25 and 1 mg/mL. SEM images revealed structural and morphological changes in eggs treated with both compounds. After 12 h of exposure to aminochalcone 1 (1.25 mg/mL), all adult nematodes were immobile, and wrinkling of the cuticle was observed. These findings indicate the ovicidal effect of aminochalcones and the inhibition of worm motility by aminochalcone 1. Our preliminary study demonstrated, for the first time, the anthelmintic activity of this class of compounds against gastrointestinal nematodes of small ruminants and suggest further anthelmintic evaluation.

## Resumo

O objetivo deste estudo foi sintetizar e avaliar o efeito *in vitro* de aminochalconas contra *Haemonchus contortus*. Aminochalconas 1 e 2 foram sintetizadas usando condensação de Claisen-Schmidt e caracterizadas usando espectroscopia de infravermelho com transformada de Fourier, ressonância magnética nuclear e espectrometria de massa. A atividade de ambas as aminochalconas foi avaliada no teste de eclosão de ovos e a da aminochalcona 1 foi avaliada posteriormente no teste de motilidade de nematódeos adultos utilizando *H. contortus* multiresistente. As estruturas químicas dos compostos sintetizados foram confirmadas e as alterações induzidas em ovos e adultos foram avaliadas usando microscopia eletrônica de varredura (MEV). As aminochalconas 1 e 2 inibiram a eclosão larvar em 98,70 e 99,89%, respectivamente, nas concentrações de 0,25 e 1 mg/mL. Imagens de MEV revelaram mudanças estruturais e morfológicas em ovos tratados com ambos os compostos. Após 12 h de exposição à aminochalcona 1 (1,25 mg/mL), todos os nematódeos estavam imóveis e foi observado enrugamento da cutícula. Esses achados indicam o efeito ovicida das aminochalconas e a inibição da motilidade dos nematódeos pela aminochalcona 1. Nosso estudo demonstrou, pela primeira vez, a atividade anti-helmíntica dessa classe de compostos contra nematódeoss gastrointestinais de pequenos ruminantes e sugere adicional avaliação anti-helmíntica.

## Introduction

Haemonchosis is caused by *Haemonchus contortus*, a hematophagous nematode that inhabits the abomasum of small ruminants, leading to decreased growth and fecundity rates and increased mortality (Flay et al., 2022). This parasite is one of the most prevalent gastrointestinal nematodes (GIN) in herds grown under grazing conditions in the subtropical and tropical regions, leading to significant economic losses in the livestock sector (Besier et al., 2016).

GIN control involves the use of commercial anthelmintics. However, the effectiveness of these drugs has declined due to the accelerated development of anthelmintic resistance (Ploeger & Everts, 2018). To this end, several studies have investigated and demonstrated the anthelmintic effects of plant-derived products, such as essential oils, extracts, and bioactive compounds (Katiki et al., 2017; Carlis et al., 2019; Davuluri et al., 2019; Aguiar et al., 2022; Barbosa et al., 2023).

Chalcones are aromatic ketones with an α, β-unsaturated carbonyl system joining two aromatic rings. They belong to a class of open-chain flavonoids (Jasim et al., 2021) and are present in several plant genera. They are extensively used in traditional medicine for therapeutic purposes and structural modifications have been shown to confer unique biological properties to chalcones, potentially enhancing their interactions with specific molecular targets (Zhou & Xing, 2015). The biological properties of chalcones include antibacterial, antifungal (Abreu et al., 2024) and antiparasitic (Bezerra et al., 2023) activities. The activity of hybrid benzimidazolyl-chalcone derivatives was *in vitro* evaluated against *H. contortus* larvae and some compounds demonstrated interesting effect (Ouattara et al., 2011). In addition, *in vitro* activity of two ferrocenyl chalcones designed (2*E*)-1-ferrocenyl-3-(3-chlorophenyl)prop-2-en-1-one and (2*E*)-1-ferrocenyl-3-(3-methylphenyl)prop-2-en-1-one against *H.contortus* larvae has been confirmed (Vázquez-Bravo et al., 2020). Furthermore, chalcone designed 1,3-diaryl-2-propen-1-one *in vitro* inhibited larval motility and development of *H. contortus* (Taki et al., 2021).

Aminochalcones constitute an important class of chalcones that contain an –NR_2_ group on either ring A or ring B (Irfan et al., 2020). However, their effect on *H. contortus* eggs and adults has not yet been demonstrated to date. Thus, the aim of this study was to synthesize, to evaluate the *in vitro* efficacy of aminochalcones against *H. contortus* eggs and adults and to verify their effects on worm morphology.

## Material and Methods

### Synthesis and characterization of aminochalcones

The aminochalcones,viz. (2E)-1-(4'-Aminophenyl)-3-(4-Dimethylaminophenyl)-prop-2-en-1-one **(aminochalcone 1)** and (2E,4E)-1-(4-Aminophenyl)-5-phenylpenta-2,4-dien-1-one **(aminochalcone 2)** were synthesized by Claisen–Schmidt condensation under basic conditions (Bhat et al., 2005). A solution of *p*-aminoacetophenone (2 mmol) in ethanol (5 mL) was added to a solution of benzaldehyde (2 mmol) in ethanol (5 mL) containing 10 drops of 50% (v/v) sodium hydroxide, and the resulting mixture was stirred for 48 h. The mixture was filtered under vacuum, washed with cold water to pH 7.0 and analyzed using thin-layer chromatography (TLC). The compounds (aminochalcones 1 and 2) were characterized using Fourier transform infrared (FT-IR), nuclear magnetic resonance (NMR) and mass spectrometry (MS).

### Anthelmintic assays

#### Recovery of *H. contortus* eggs and adults

A lamb was housed in a metabolic cage and treated with 5 mg/kg of levamisole (Ripercol^®^, Zoetis, Brazil), 0.2 mg/kg of ivermectin (Ivomec^®^, Boehringer-Ingelheim, Brazil), and 2.5 mg/kg of monepantel (Zolvix^®^, Novartis, Brazil), single dose on alternate days (Barbosa et al., 2023). After total clearance of natural GIN infection, confirmed by fecal egg counts (FEC) and coproculture, the lamb was uniquely infected by oral inoculation withthird-stage larvae (L3) of *H. contortus*. *Haemonchus contortus* Kokstad (KOK) isolate was used as a reference because it is resistant to benzimidazoles, levamisole, and macrocyclic lactones. The isolate was provided by Institut National de Recherche pour l'Agriculture, l'Alimentation et l'Environnement (INRAE), Nouzilly, France. The choose of aminochalcones concentrations used in the anthelmintic assays was based on their solubility and findings of others studies with active compounds.

#### Egg hatching test (EHT)

The EHT was performed according to the method described by Coles et al. (2006). To recover *H. contortus* eggs, feces were collected directly from animal harboring a monospecific infection with the KOK isolate and processed according to the technique described by Hubert & Kerboeuf (1992). Briefly, 250 μL of an egg suspension containing approximately 100 fresh eggs was incubated for 48 h at 27 ˚C with 250 μL of aminochalcone 1 in the concentration range of 0.25–0.015625 mg/mL or aminochalcone 2 in the concentration range of 1.0–0.0625 mg/mL. Lugol's iodine solution was added to stop larval hatching, and the eggs and first-stage larvae (L1) were counted under a light microscope. This test was conducted with two controls: 3% Tween (Sigma-Aldrich^®^) in phosphate-buffered saline (PBS) was the negative control and 0.1 mg/mL thiabendazole (Sigma-Aldrich^®^) was the positive control. Three repetitions with five replicates for each treatment and control group were performed.

#### Adult worm motility test (AWMT)

AWMT was performed according to the method described by Hounzangbe-Adote et al. (2005). The experimentally infected lamb was euthanized by chemical method. For this, the sedation was followed by general anesthesia with thiopental. After confirmation of animal unconsciousness and loss of reflex, potassium chloride was administrated. To obtain the parasites, the abomasum was removed, opened, and placed in a saline solution maintained at 37 ˚C. Females adult *H. contortus* were quickly collected and placed into 24-well plates at a density of three worms/plate (in 500 μL of PBS enriched with 4% penicillin/streptomycin (Sigma-Aldrich^®^) at 37 ˚C). After incubation for 1 h (37 ˚C, 5% CO_2_), 500 μL of aminochalcone 1 was placed in each well at the following concentrations: 2.5 to 0.15625 mg/mL. The negative control was 3% Tween in PBS. After incubation for 3, 6, 9, and 12 h, the motility and survival of the adult worms were observed under an inverted microscope. Survival or death of the parasite was confirmed by a soft touch using a syndesmotome. Five replicates were used for each treatment and control group.

#### Scanning electron microscopy (SEM) processing of eggs and adults

*Haemonchus contortus* eggs exposed to the highest concentrations of aminochalcone 1 (0.25 mg/mL) and 2 (1.0 mg/mL), and the controls (negative and positive) were analyzed using SEM. At least 10 eggs showed the same pattern of change. Ten microliter samples containing eggs were deposited on aluminum stubs with double-sided carbon adhesive tape and observed. This methodology was adapted from Zarza-Albarrán et al. (2020).

The medial region of *H. contortus* adults was evaluated using SEM. Parasites exposed to 1.25 mg/mL aminochalcone 1 for 12 hours and the negative control were randomly selected and fixed in a 2.5% glutaraldehyde solution in 0.1 M sodium cacodylate buffer for 72 h. The parasites were then dehydrated in a gradual ethanol concentration system (15, 30, 50, 70, and 90% for 1 h each and 100% three times for 30 min in each treatment). After 24h, the samples were dried using a K850 critical point drying apparatus (Quorum Technologies Ltd., UK), mounted on stubs, and coated with gold-palladium (Barbosa et al., 2023).

For visualization, a microscope (FEI Company^®^ (USA) with an accelerating voltage of 15 kV was used. The magnification was set at 8.000× for eggs and 110× for adults, following which possible qualitative changes were observed.

### Statistical analysis

In the EHT, the effects of aminochalcones 1 and 2 were determined using the following equation: [number of eggs/(number of eggs + number of L1)] × 100. The motility of adult worm streated with aminochalcone 1 was evaluated according to the following formula: number of motile worms/total number of worms per well.

Initially, the data of the EHT and AWMT were analyzed and the assumptions of normality and homogeneity were satisfactorily fulfilled (p>0.05). The percentage of hatching larvae in EHT was analyzed by one-way analysis of variance (ANOVA) to evaluate the effect of each aminochalcone (1 and 2) followed by Tukey’s test using GraphPad^®^ Prism 8.0. The results of the AWMT were analyzed by ANOVA with repeated measures to evaluate the effect of aminochalcone 1 followed by Tukey’s test using SPSS^®^ 23.0 program for Windows. The effective concentrations of aminochalcones to inhibit 50% (EC_50_) of larvae hatching and adult motility were determined by the probit linear regression model using SPSS^®^ 23.0 program for Windows.

## Results

The structures of aminochalcones 1 and 2 were determined by NMR, IR, and MS as shown below.

### (2E)-1-(4’-aminophenyl)-3-(4-dimethyaminophenyl)-prop-2-en-1-one (1)

Orange solid (Yield: 35.66%), m.p. 169 - 170oC; FT-IR (KBr, νcm-1): 3500, 3460, 1610, 1570, 1350, 1160, 980. 1H-NMR (CD3OD, 300 MHz): δ 7.57 (d, H-2/H-6, J = 8.85 Hz), 6.69 (d, H-3/H-5, J = 8.76 Hz), 7.89 (d, H-2’/H-6’, J = 8.73 Hz), 6.76 (d, H- 3’/H-5’, J = 8.88 Hz), 7.46 (d, H-α, J = 15.39 Hz), 7.68 (d, H-β, J = 15.39 Hz), 3.02 (s, 2CH3). 13C-NMR (CD3OD, 75 MHz): δ C-1 124.7, C-2/C-6 131.5, C-3/C-5 113.3, C-4 153.8, 2CH3 40.4, C-1’ 128.4, C-2’/C-6’ 132.3, C-3’/C-5’ 114.6, C-4’ 155.2, C-α 117.6, C-β 145.8, C=O 190.8. MS (EI) m/z (M+. 266), calculated for C_17_H_18_N_2_O/266.

### (2E,4E)-1-(4-aminophenyl)-5-phenylpenta-2,4-dien-1-one (2)

Dark orange solid (Yield: 34%), m.p. 151,8 - 152oC; FT-IR (KBr, νcm-1): 3458, 3376, 1635, 1611, 1576 1564. 1H-NMR (CD3OD, 300 MHz): δ 7.26 – 7.35 (m, Ar), 7.84 (d, H-2`/H-6`, J = 8.79 Hz). 6.67 (d, H-3`/H-5`, J = 8.76 Hz). 7.00 – 7.25 (m, H-α, H-7, H- 8), 7.38 (ddd, H-β, J = 15.96, 8.43, 1.65 Hz). 13C-NMR (CD3OD, 75 MHz): δ C-1 138.0, C-2/C-6 128.7, C-3/C-5 130.0, C-4 128.4, C-7 126.8, C-8 142.6, C-1` 127.7, C- 2`/C-6` 132.5, C-3`/C-5` 114.6, C-4`155.6, C-α 130.5, C-β 144.6, C=O 190.3. MS (EI) m/z (M+. 253), calculated for C_17_H_15_NO/253.

The effects of aminochalcones 1 and 2 on the EHT are shown in Table 1. Aminochalcone 1 had an efficacy greater than 98% at a concentration of 0.25 mg/mL, whereas aminochalcone 2 inhibited 99% of the larvae hatching at a concentration of 1.0 mg/mL. These results did not differ significantly from those of the positive controls (p>0.05). The EC_50_ values for aminochalcones 1 and aminochalcone 2 were 0.10 mg/mL and 0.19 mg/mL, respectively, and the effects were dose-dependent.

**Table 1:** Effect (mean ± standard error of mean) of (E)-1-(4-aminophenyl)-3- (4-(dimethylamino)phenyl)prop-2-en-1-one (Aminochalcone 1) and (2E,4E)-1-(4-aminofenil)-5-fenilpenta-2,4-dien-1-one (Aminochalcone 2) on *Haemonchus contortus* egg hatching.

**Concentrations (mg/mL)**	**Chalcone 1**	**Concentrations (mg/mL)**	**Chalcone 2**
-	-	1.0	99.47 ± 0.1^A^
-	-	0.5	92.10 ± 0.6^A^
0.25	98.51 ± 0.8^A^	0.25	69.02 ± 2.8^B^
0.125	72.80 ± 7.3^B^	0.125	41.09 ± 3.2^C^
0.0625	23.42 ± 2.9^C^	0.0625	18.50 ± 4.2^D^
0.03125	5.09 ± 0.5^D^	-	-
0.015625	3.9 ± 0.3^D^	-	-
Negative control*	3.3 ± 0.2^D^	Negative control*	3.6 ± 0.2^E^
Positive control**	95.57 ± 1.2^A^	Positive control**	97.64 ± 0.3^A^

Negative control*: Tween.

Positive control**: Thiabendazole.

Capital letters compare the mean values in the columns for each aminochalcone.

Different letters indicate significantly different values (p<0.05).

The effect of aminochalcone 1 on *H. contortus* motility is shown in Table 2. Aminochalcone 1 reduced worm motility in a concentration and time-dependent manner. At the highest concentration tested (2.5 mg/mL), aminochalcone 1 treatment led to a rapid reduction in worm motility at the first observation (3 h post treatment). Total inhibition of worm motility was also observed at concentrations of 1.25, 0.625, and 0.3125 mg/mL after 6, 9, and 12 h of exposure, respectively. The EC_50_ value was 0.17 mg/mL at 12 h post-exposure. Worm motility in the negative control (Tween 80^®^) was 100% 12 h post-exposure.

**Table 2:** Effect (mean ± standard error of mean) of (E)-1-(4-aminophenyl)-3-(4-(dimethylamino)phenyl)prop-2-en-1-one (Aminochalcone 1) on inhibition of adult *Haemonchus contortus* motility.

**Concentrations (mg/mL)**	**% worms showing mortality post-exposure to treatments**			
	**3 h**	**6 h**	**9 h**	**12 h**
2.5	100.0 ± 0.0^Aa^	100.0 ± 0.0^Aa^	100.0 ± 0.0^Aa^	100.0 ± 0.0^Aa^
1.25	95.0 ± 5.0^Aa^	100.0 ± 0.0^Aa^	100.0 ± 0.0^Aa^	100.0 ± 0.0^Aa^
0.625	0.0 ± 0.0^Ba^	80.0 ± 8.2^Bb^	100.0 ± 0.0^Ac^	100.0 ± 0.0^Ac^
0.3125	0.0 ± 0.0^Ba^	46.7 ± 22.6^Cb^	66.7 ± 8.7^Bc^	100.0 ± 0.0^Ad^
0.15625	0.0 ± 0.0^Ba^	0.0 ± 0.0^Da^	20.0 ± 23.57^Cb^	20.0 ± 13.3^Bb^
Negative control*	0.0 ± 0.0^Ba^	0.0 ± 0.0^Da^	0.0 ± 0.0^Da^	0.0 ± 0.0^Ca^

Negative control*: Tween.

Capital letters compare the mean values in columns, and small letters compare the mean values in lines.

Different letters indicate significantly different values (p<0.05).

**Figure 1:**
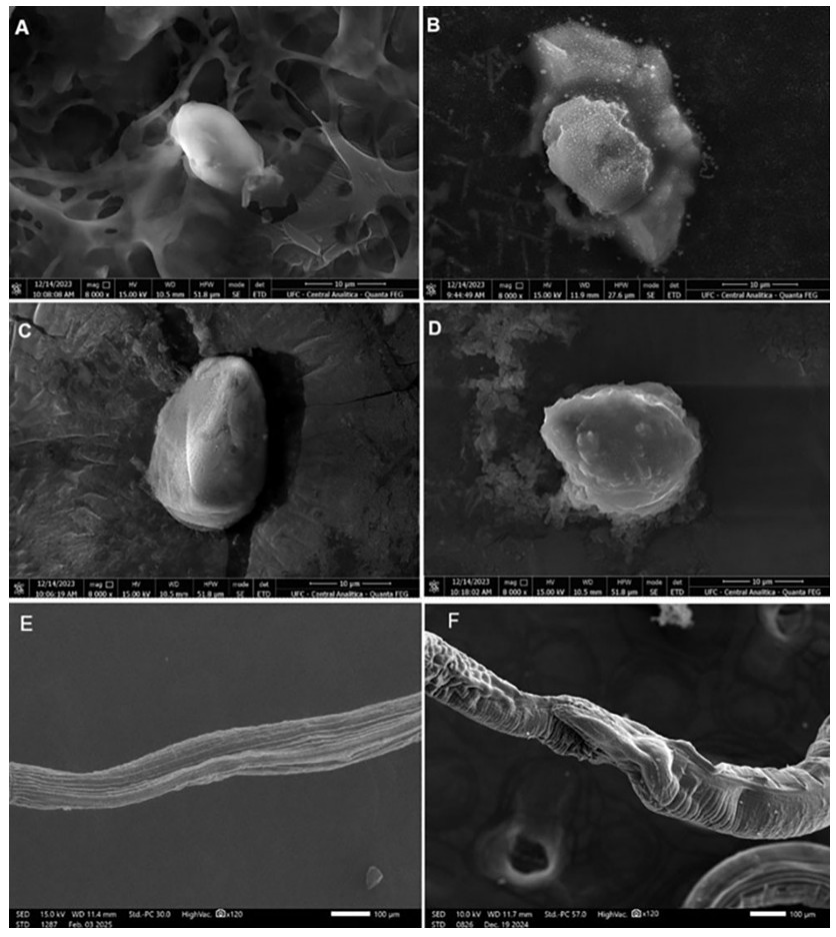
*Haemonchus contortus* treated with aminochalcones and controls observed under a scanning electron microscope. (A) Eggs - Negative control (Tween); (B) Eggs - Positive control (thiabendazole); (C) Eggs - Aminochalcone 1 (0.25 mg/mL); (D) Eggs - Aminochalcone 2 (0.25 mg/mL); (E) Adults - Negative control (Tween); (F) Adults - Aminochalcone 1 (1.25 mg/mL).

Figure 1 shows *H. contortus* eggs and adults after treatment with synthetic aminochalcones and controls. In the negative control (Figure 1A), the eggs exhibited intact morphology and a smooth eggshell surface. Treatment with both aminochalcones (Figures 1C and 1D) resulted in the loss of eggshell morphology, including cracks in the morula and formation of intracellular spaces, similar to the effect observed in the positive control (thiabendazole) (Figure 1B). SEM micrographs also showed alterations in adult *H. contortus* after *in vitro* exposure to aminochalcone 1 (Figure 1F). The main change observed was wrinkling of the longitudinal cuticular rigdes. The longitudinal cuticular ridges well preserved in the negative control group (Figure 1E).

## Discussion

Currently, only a few classes of anthelmintic drugs are commercially available, including benzimidazoles, imidazothiazoles, tetrahydropyrimidines, macrocyclic lactones, amino acetonitrile derivatives, and salicylanilides. However, anthelmintic resistance in nematode populations is a serious global problem (Ploeger & Everts, 2018). Due to the increasing prevalence of anthelmintic resistance, there is an urgent need for concerted efforts to identify novel strategies for the development of new effective drugs.

In the present study, we investigated the potential anthelmintic effects of aminochalcones on *H. contortus* eggs and adults resistant to synthetic anthelmintics. To the best of our knowledge, this is the first study to assess the activity of aminochalcones against *H. contortus* eggs and adults.

Aminochalcones 1 and 2 demonstrated promising efficacy in the EHT. The ovicidal effect of aminochalcone 1 (EC_50_ = 0.10 mg/mL) and aminochalcone 2 (EC_50_ = 0.19 mg/mL) against *H. contortus* was superior to other synthethic compounds, such as linalool (EC_50_ = 0.29 mg/mL), eugenol (EC_50_ = 0.57 mg/mL), vanilin (EC_50_ = 0.57 mg/mL), cineole (EC_50_ = 4.74 mg/mL) and limonene (EC_50_ = 207.56 mg/mL) (Katiki et al., 2017). Both aminochalcones also showed ovicidal efficacy against *H. contortus* superior to that of other open-chain flavonoids. Zarza-Albarrán et al. (2020) evaluated galloyl flavonoids from *Acacia farnesiana* and demonstrated their activity against *H. contortus* eggs. The ethyl acetate fraction of the hydroalcoholic extract showed the best ovicidal effect, with EC_50_ of 0.51 mg/mL. Furthermore, luteolin and 4′-O-methylcatechin exhibited no ovicidal activity against *H. contortus* (Delgado- Núñez et al., 2020).

The ovicidal effect of aminochalcones evaluated in our study can be a consequence of the presence of an α,β-unsaturated double bond. The α,β-unsaturated functionality is one of the most reactive substructures commonly found in both synthetic and natural molecules, responsible for pharmacological activities (Arshad et al., 2017). The higher number of nitrogen atoms in the chemical structure of aminochalcone1 may have contributed to its superior efficacy; therefore, its effect was evaluated in the AWMT. However, this is a preliminary study and pharmacological activity of aminochalcones should be clarified in future studies.

SEM images of *H. contortus* eggs following treatment with aminochalcones 1 and 2 revealed alterations in eggshell morphology. Our results corroborated those of Delgado-Núñez et al. (2020), who verified the significant disruption of cellular architecture, including cracks in the morula and formation of intracellular spaces, in *H. contortus* eggs exposed to isorhamnetin, a flavonoid from *Prosopis laevigata* leaves. Further, according to Zarza-Albarrán et al. (2020), SEM analyses of *H. contortus* eggs exposed to organic fractions from *A. farnesiana* showed the presence of granular structures attached to the eggshell.

The AWMT is used to evaluate the *in vitro* action of natural and synthetic compounds and can be used in tandem with SEM to assess changes in the morphology of adult nematodes (Aguiar et al., 2022; Barbosa et al., 2023). Herein, aminochalcone 1 completely inhibited the motility of *H. contortus* at the highest concentrations tested (2.5 to 0.625 mg/mL). Turani et al. (2024) synthesized a series of chalcones using a biochar-derived catalyst obtained from the pyrolysis of tree-pruning waste, resulting in a library compatible with various functional chemical groups. The effects of these compounds against *C. elegans* adults, a nematode widely used as a model organism for anthelmintic drug discovery, were evaluated. Among the tested chalcones, compound 3 exhibited the highest efficacy. The synthesis of this compound involved the use of benzaldehyde and acetophenone, which were also used in our experiments for the synthesis of aminochalcone 1. The similar chemical structure can explain the efficacy of aminochalcone 1 against *H. contortus* adults.

Furthermore, aminochalcone 1 demonstrated superior efficacy against motile worms compared to the other investigated flavonoids. Davuluri et al. (2019) evaluated the efficacy of flavonoid-rich extracts from *Anacardium occidentale* and *Artocarpus heterophyllus* against *H. contortus* adults. The EC_50_ values were 1.04 and 2.39 mg/mL, respectively. This suggests that aminochalcone 1 has good potential in reducing adult motility, further reinforcing its role as an effective alternative to other flavonoid-based treatments. The finding of compounds with promising *in vitro* effects on the adult is important because this stage is the pathogenic form of *H. contortus*. The adult is responsible for the clinical signs and reduced productivity observed inhosts.

The position of the substituents on the chalcone rings plays an essential role in their pharmacological activities. Chalcones designated as 4 and 5 by Turani et al. (2024) exhibited lower efficacy against *C. elegans* than chalcone 3 and its methoxy-containing analog (chalcone 6). The presence of an amino group at the *para* position of aminochalcone 1 may have contributed to its efficacy against *H. contortus* adults. Electron-donating groups, such as amino groups, increase the electronic density of the molecule, favoring molecular interactions with biological targets (Irfan et al., 2020), such as enzymes present in the nematode cuticle.

Aguiar et al. (2022) also demonstrated wrinkling of the longitudinal cuticular ridges in *H. contortus* adults treated with carvone. Carlis et al. (2019) verified the structural changes induced in larvae isolated from L3 migration inhibition test performed on *Piper cubeba*, and the main changes observed were associated with the cuticle. Based on these experimental results, phosphomethyltransferase (PMT) was selected by Borges et al. (2023) as the molecular target for *in silico* studies. These authors demonstrated that hinokinin, a lignan from *P. cubeba*, could inhibit PMT-2 by altering the synthesis of phosphocholine, consequently affecting the cuticle of *H. contortus* larvae. Since aminochalcone 1 also affected the cuticle of *H. contortus* in this current study, *in silico* studies should be performed to evaluate its interaction with PMT. These aforementioned studies have contributed to the search for promising new targets for the development of agents with anthelmintic properties.

This study indicated that aminochalcones have ovicidal activity against *H. contortus* and that aminochalcone 1 has superior inhibitory effects on larvae hatching, including inhibition of adult worm motility. Alterations in eggshell morphology and disruptions in nematode cuticle integrity were observed, suggesting that these compounds affected the structural integrity of *H. contortus*. These findings indicate that aminochalcones 1 and 2 are promising candidates for the development of novel anthelmintic agents. Future studies should investigate their *in vivo* efficacy, safety, and pharmacokinetics to validate their therapeutic potential.

## Data Availability

Data will be made available on request.
